# Prevalence, predisposition and prevention of type II diabetes

**DOI:** 10.1186/1743-7075-2-29

**Published:** 2005-10-18

**Authors:** Dong Cheng

**Affiliations:** 1 Department of Obesity and Metabolic Research, Pharmaceutical Research Institute, Bristol-Myers Squibb Company, Princeton, NJ 08543, USA

## Abstract

In 2000, more than 151 million people in the world are diabetic. It is predicted that by 2010, 221 million people and by 2025, 324 million will be diabetic. In the U.S., for the population born in 2000, the estimated lifetime risk for diabetes is more than 1 in 3. The economic and human cost of this disease is devastating. The current cost of diabetes in the U.S. is estimated to be at $132 billion, which includes $92 billion of direct medical costs and $40 billion of indirect costs such as disability, work loss and premature mortality. The outbreak of the current diabetic epidemic has been accompanied by a similarly drastic increase in obesity. The relation between the two is a matter of debate but presumably both are caused by changes in dietary habits and an increasingly sedentary modern lifestyle. Compelling scientific evidence indicates that lifestyle modification effectively prevents or delays the occurrence of type 2 diabetes. Recent clinical trials also demonstrate that success in the treatment of obesity, either surgically or pharmacologically, leads to the prevention of type 2 diabetes among the obese. Clinical data have also revealed that the insulin sensitizing agent troglitazone is efficacious in both β-cell preservation and delaying the onset of type 2 diabetes. Future safe and more effective anti-obesity medicines and insulin sensitizing agents that help to preserve β-cell function, in addition to efforts of lifestyle modification, thus hold promise for the overweight population with potential for reduction in the development of diabetics.

## Background

Social affluence is a double edged sword. On the one hand, life is more convenient than ever because of the advances in technology; on the other hand, the incidence of diabetes is occurring at an alarming rate. The explosive increase in number of people diagnosed with diabetes makes this disease a new health threat in the 21^st ^century. Understanding the etiology of and finding a way to prevent diabetes, especially type 2 diabetes, is an urgent challenge for the health care community and our society.

## Epidemics of type 2 diabetes

In 2002, more than 18 million Americans, about 6.9% of the U.S. population, have diabetes [1]. Globally, the number of people that has been diagnosed with diabetes has also exploded in the past two decades. In 2000, 151 million people in the world were diabetic. With the current rate of increase, it has been projected that 221 million people will be diabetic in 2010 and 324 million by 2025 [2].

There are two major forms of diabetes: type 1 and type 2 diabetes [3]. The hall mark of type 1 diabetes is the destruction of insulin producing β-cells in the pancreas, primarily due to autoimmune responses. Type 1 diabetes is manifested with absolute insulin deficiency. In contrast, type 2 diabetes is characterized by two defects: insulin deficiency and insulin resistance. Type 2 diabetes accounts for 90 to 95% of the incidence of diabetes. The current epidemic outbreak of diabetes reflects the high prevalence of type 2 diabetes.

While the seriousness of the epidemic was only fully recognized recently, the threat by a trend of the increase of incidences in diabetes was first recognized by Elliott Joslin some 80 years ago [4]. From a historical perspective, the epidemic of type 2 diabetes today has developed steadily through the decades in the last century. According to the data of National Health Interview Survey, the incidence in 1990–1992 was 6.4 times the rate of 1935–1936 [5]. In a 10-year span from 1990 to 1999, the prevalence further increased by 40% from 4.9% to 6.9% [6]. Assuming the rate of increase in incidences continues, the data from the National Health Interview Survey (1984–2000) predicts that the residual lifetime risk of diabetes for individuals born in 2000 is 32.8% for males and 38.5% for females. The highest risk for ethnic subpopulations is in Hispanic females whose lifetime risk of becoming diabetic is 52.5% [6].

The global statistics indicate that the burden of type 2 diabetes is not restricted to the developed nations, but also is a problem for developing countries. For example, in the Pacific island of Nauru, type 2 diabetes is present in about 40% of adults [7]. Ironically this disease was not present a half century ago in this region. In a 11-year follow-up study in Mauritius, the prevalence of type 2 diabetes, in both men and women, regardless of ethnic group, increased steadily from 12.8% in 1987, to 15.2% in 1992, and to 17.9% in 1998 [8]. In recent years, the prevalence of type 2 diabetes has also increased in world's two largest populated nations, China [9] and India [10]. India now has the most people – 38 million – with diabetes. China is ranked second and has 23 million diabetics. By 2025, these numbers are expected to be doubled to about 73 million and 46 million, respectively [11].

Although previously type 2 diabetes was predominantly diagnosed in middle-aged or older people, the age of onset of this disease has decreased. Japan has seen an approximately 4-fold rise in the incidence of type 2 diabetes in 6- to 15-year-olds. This rate now is outnumbering that of type 1 diabetes in that country [11]. Data from the U.S. indicates that 8–45% of recently diagnosed cases of diabetes in the young is due to type 2 diabetes [12].

Uncontrolled diabetes leads to other serious medical complications including a substantial increase in premature morbidity and mortality [13,14]. It is reported that the incidence of cardiovascular disease among adult diabetics is 37.2%, which is much higher than the incidence in the general population [14]. Prevalence of ischemic heart disease among the diabetics 18 to 44 years of age is 14 times for those without diabetes [14]. The high blood glucose of diabetes also causes both micro and macro vascular damages. Damages in large vessels lead to stroke and cardiovascular complications, whereas damages to vessels in the extremities, eyes and kidneys lead to amputation, blindness and kidney failure. As a result, each year, as many as 24,000 diabetics in the U.S. become blind [14]; more than 100,000 require kidney dialysis or kidney transplantation which accounts for more than 40% of new cases of end-stage renal disease. Furthermore, 82,000 diabetics need to have a toe or a leg amputated, accounting for more than half of all non-traumatic lower-extremity amputations in the U.S. [14]. The diabetes epidemic is thus a large assault on the health care system and a huge burden for the society. The current cost of diabetes in the U.S. is estimated as $132 billion, which includes direct medical costs of $92 billion and indirect costs (disability, work loss, premature mortality) of $40 billion [1].

## Obesity, Metabolic Syndrome, prediabetes

### Obesity

What is the driving force for the current worldwide epidemic of diabetes? Environmental factors such as adoption of a sedentary lifestyle, changes in eating habits and consequent obesity, are likely the main causes or at least a parallel problem. This hypothesis is supported strongly by the studies in migrating populations. For example, the prevalence of diabetes in the urban regions of India is increasing dramatically in affluent migrant Indians [15]. Rates among Asian Indians in countries such as South Africa, the U.K. and Fiji are much higher than reported in most parts of India itself [16]. The incidence is also rising among Africans who have either urbanized or immigrated to the U.S. [17,18]. Before the 1990s, the rates of type 2 diabetes in Japan were quite low; but high rates were found in Japanese living in the U.S. [16]. Rates of increased incidence of diabetes in populations of Chinese origin also vary with environment changes. In the Northern part of mainland China, in the age group 30 to 64, about 1% was the rate for type 2 diabetes; this number went to 4 to 5% among the Chinese in Singapore and to 11 to 12% in Mauritus [19]. Many native American Indian tribes have a higher prevalence of diabetes as compared with the general U.S. population; but Eskimos appears to be an exception, whose diabetes rate is not distinct from white Americans [20]. All these population-based studies reveal a similar pattern: environmental changes and adaptation of sedentary lifestyle, resulting from industrialization and migration to urban cities, lead to the development of type 2 diabetes.

Parallel to the increase of incidences in type 2 diabetes is the increase of obesity in the population. According to the definition recommended by the World Health Organization (WHO) expert committee for the classification of overweight and obesity, today, close to 65% of the U.S. adult population is overweight, and among them, above 30% are obese [21]. In this classification, Body Mass Index (BMI) between 25 to 30 is considered as overweight and BMI above 30 is considered as obese. Based on the statistics collected by National Health and Nutrition Examination Survey (NHANES), in a representative sample of the U.S. population, the prevalence of an overweight condition has increased by 40% (from 46% in the period of 1976–1980 to 64.5% in the period of 1999–2000), and the prevalence of obesity has increased by 110% (from 14.5% to 30.5%) [21,22]. In addition, the increase in weight in the young population also exhibits an alarming growth. If overweight is defined as at or above the 95th percentile of the sex-specific BMI for age growth charts, among those aged 2 through 19 years, the prevalence of overweight was 15.5% among 12-through 19-year-olds, 15.3% among 6-through 11-year-olds, and 10.4% among 2-through 5-year-olds according to 1999–2000 NHANES data. These numbers are significantly increased from 10.5%, 11.3%, and 7.2%, respectively, as collected in 1988–1994 (NHANES III).

Despite the on-going debate as to whether obesity should be labeled as a disease, obesity, directly and indirectly, has become the global health challenge. It has been estimated that close to 300,000 deaths each year in the U.S. may be attributable to obesity [23]. In a study comparing health care spending on obese and normal-weight Americans between 1997 and 2001, it was found that per capita spending for the obese was more than $1000, or 37%, higher than spending on normal-weight people in 2001 [24].

Obesity has become a burden for society, as it is a major cause of lost productivity. The financial burdens that are consequences of obesity are associated with the treatment needed for the accompanying disease states, such as cardiovascular disease, cancer, hypertension, and most intimately, to type 2 diabetes [25]. In a 16-year follow-up study of 84,941 women, it was documented that 3300 new cases of type 2 diabetes were diagnosed from 1980 to 1996 [26]. Among these cases, body weight was the single most important predictor of diabetes. As high as ~80% of the cases of type 2 diabetes could be attributed to the combined effect of inactivity and high body weight [27].

In the realm of lifestyle, the change of dietary composition, in addition to the total caloric intake, is probably another important factor that is implicated in the epidemics of type 2 diabetes. For example, one important but not well-appreciated dietary change has been the substantial increase in refined carbohydrates especially simple carbohydrate from high intake of sucrose and high fructose corn syrup, a common sweetener used in the food industry. High influx of carbohydrate is predicted to drive the increase of de novo hepatic lipogenesis and perturbs the glucose homeostasis that appear to underlie the induction of insulin resistance [28,29].

The exact molecular and cellular connection between obesity and type 2 diabetes has not been entirely explained. In particular, there is no unifying hypothesis that explains the various states of "garden-variety" insulin resistance associated with diet-induced obesity. One of the hypotheses highlights the pathological roles of lipid abnormality accompanying obesity or high body weight, postulates that accumulation of fatty acids or fatty acid derivatives in muscle and liver produce insulin resistance [30]. At the cellular level, it has been proposed that the accumulation of diacylglycerol might activate protein kinase C (PKC) (probably by PKC-θ in rodents and by PKC-β or -δ in humans) [31]. The activated PKC in turn phosphorylates and activates other serine kinases such as IKK-β[32]and JNK-1 [33], leading to phosphorylation of serine sites on IRS-1 insulin receptor substrate (IRS) 1 and 2 at Serine/Threonine sites, blunting the insulin signaling pathway[34]. It is also suggested that ceramide, a down stream product of fatty acid metabolism, down regulates insulin signaling in muscle [35]. It has been shown that ceramide blocks insulin-stimulated tyrosine phosphorylation of IRS-1 and its subsequent recruitment and activation of PI3 kinase. The abnormalities in fat metabolism and insulin resistance seem to form a vicious cycle. Hyperinsulemia from elevated caloric or carbohydrate intake especially in the presence of high fat may cause insulin resistance in adipocytes leading to elevated fatty acids which, in turn may cause insulin resistance in muscle. At the same time, insulin resistance further exacerbates the abnormalities in hepatic fat metabolism [36,37]. In addition to the adverse metabolic consequences in insulin sensing tissues, fat accumulation also has harmful effects in insulin producing β-cells. The increased tissue levels of fatty acyl CoA cause β-cell abnormalities in nondiabetic obese patients and ultimately result in obesity-dependent diabetes [38]. Of note, these potential mechanisms are not mutually exclusive. In fact, they could all play roles at various stages for the development of type 2 diabetes, a long and gradual process [39].

### Metabolic Syndrome

Obesity and insulin resistance often coexist along with other abnormalities such as hypertension and dyslipidemia. In 1988, Reaven introduced the concept of Metabolic Syndrome X in the Banting Medal address, in order to emphasize the coexistence of multiple metabolic abnormalities. This term underscores the fact that insulin resistance and its compensatory hyperinsulinemia develop with dysregulation of glucose and lipid metabolism [40]. The dysregulation of glucose metabolism is represented by varying degree of glucose tolerance. The dysregulation of metabolism of lipid is manifested by the increase of plasma triglyceride levels and the decrease of plasma HDL cholesterol. Numerous population-based studies have described the characteristics of Metabolic Syndrome X. Considerable information has evolved from the original recognition of the clustering of metabolic abnormalities centering around insulin resistance/hyperinsulinemia. The original term Metabolic Syndrome X has become a synonym of Insulin Resistance Syndrome or Metabolic Syndrome. Currently, the abnormalities clustered with insulin resistance include some degree of glucose intolerance (either manifested as elevated fasting glucose or impaired glucose tolerance), dyslipidemia (elevated triglycerides, reduced HDL-c, reduced LDL-particle diameter), endothelial dysfunction (increased mononuclear cell adhesion, cellular adhesion molecules and decrease in endothelial-dependent vasodilatation), increased procoagulant factors, and elevation of inflammation [41].

The Third Report of the National Cholesterol Education Program Expert Panel on Detection, Evaluation, and Treatment of High Blood Cholesterol in Adults (Adult Treatment Panel III) (ATP III) has provided a working definition of the syndrome for the first time. Individuals having three or more of the following criteria were defined as having the metabolic syndrome: 1) abdominal obesity: waist circumference > 102 cm in men and > 88 cm in women; 2) hypertriglyceridemia: ≥ 150 mg/dL; 3) low HDL cholesterol: < 40 mg/dL in men and < 50 mg/dL in women; 4) high blood pressure: ≥ 130/85 mm Hg; 5) high fasting glucose: ≥ 110 mg/dL [42]. World Health Organization has defined metabolic syndrome in the following way: at least 1 of abnormalities in glycemic metabolism (type 2 diabetes, impaired glucose tolerance, insulin resistance) accompanied by at least 2 other conditions: high blood pressure (≥ 140/90 mm Hg), obesity, dyslipidemia (hypertriglyceridemia or low HDL) and microalbuminuria [43]. (For comparison of the differences between the two definitions, see Table 1). The subtle differences among various definitions do not change the overall conclusion that the prevalence of metabolic syndrome is high. It has been estimated, using ATP III criteria, that the prevalence in the U.S. population was 21.8% between 1988–1994. Using 2000 census data, about 47 million U.S. residents are estimated to have the metabolic syndrome [44].

**Table 1:** Definition of the Metabolic Syndrome.

**ATP III Definition**
Any three or more of the following criteria:
a) Waist circumference: >102 cm in men, >88 cm in women
b) Serum triglycerides: ≥ 150 mg/dL
c) HDL-cholesterol: <40 mg/dL in men, < 50 mg/dL in women
d) Blood pressure: ≥ 130/85 mm Hg
e) Serum glucose: >110 mg/dL
**WHO Definition**
Diabetes or IFG or IGT or insulin resistance, plus at least two of the following criteria
a) Waist-to-hip ratio: >0.90 in men, >0.85 in women
b) Serum triglycerides: >150 mg/dL par or HDL-cholesterol: <35 mg/dL in men and <40 mg/dL in women
c) Blood pressure: >140/90 mmHg
d) Urinary albumin excretion rate > 20 ug/min or albumin/creatinine ratio >30 mg/g

Since the introduction of the concept of Metabolic Syndrome and the subsequent accumulation of large body of research data, it is beyond doubt that metabolic abnormalities in the areas of carbohydrate metabolism, body weight homeostasis, lipid metabolism and blood pressure regulation, tend to occur concomitantly. However, the precise criteria for the definition of Metabolic Syndrome has yet to be unified. This is probably due to the enormous complexity of physiological/pathological conditions that Metabolic Syndrome covers. As such, the clinical utility and value of diagnosing Metabolic Syndrome is debated [45,46]. In a recent Joint Statement from the American Diabetes Association and the European Association, it is stated that "Our analysis indicates that too much critically important information is missing to warrant its designation 'syndrome'. Until much needed research is completed, clinicians should evaluate and treat all CVD risk factor without regard to whether a patient meets the criteria for diagnosis of the 'metabolic syndrome"'[47].

### Prediabetes

Epidemiological studies indicate that the development of type 2 diabetes takes place over a long period of time from the initial decline of insulin effectiveness ultimately progressing to frank diabetes when β-cell function collapses. In most patients, insulin resistance can be detected long before the deterioration of glucose intolerance occurs. Approximately 5 to 10% of glucose-intolerant patients progress to frank type 2 diabetes in a given year. Inasmuch as Metabolic Syndrome emphasizes the condition of insulin resistance, the syndrome itself is not type 2 diabetes, but a large percentage of the people with Metabolic Syndrome will develop type 2 diabetes if the condition of insulin sensitivity is not improved. While the confident diagnosis of Metabolic Syndrome is technically difficult because the criteria are elusive, a simpler term – prediabetes – was introduced to define patients who have modestly higher glucose levels than normal but have not yet reached diabetic glucose levels. There are two criteria for prediabetes: impaired glucose tolerance (IGT) or impaired fasting glucose (IFG). The American Diabetes Association (ADA) specifies as a 2-h postprandial glucose 140 – 199 mg/dL as IGT, and fasting plasma glucose 110 – 125 mg/dL as IFG [48]. In 2005, in the U.S. alone, 41 million adults are reported to have prediabetes [49].

The big question is when the diabetes clock starts ticking. For eye or small vessel complication, it might be the consequence of increased plasma glucose level. For cardiovascular disease, however, the risk likely starts in the prediabetes stage. Subjects with IFG have been demonstrated to have an increased risk for macrovascular disease, in a variety of population-based studies [50,51]. Thus insulin resistance has been considered as an independent risk factor for the cardiovascular disease. Because of this, the detection of IGT is of great importance for general public health, in particular to help prevent cardiovascular complications in the high risk population [52]. Although the definition of prediabetes is simpler than Metabolic Syndrome, the glucose tolerance test remains a time-consuming method. IFG can not replace the tolerance test, since each test may reflect different defective conditions of glucose metabolism. Because the glucose tolerance test is difficult to implement as a population screen test, a large number of people have been left undiagnosed and unaware of the risks they face. This is particularly true for people who are overweight. Based on NHANES III data, among the overweight adults in the U.S., 17.1% aged 45–74 years had IGT, 11.9% had IFG, 22.6% had prediabetes, and 5.6% had both IGT and IFG. It is estimated that in the year 2000, 9.1 million overweight adults aged 45–74 had IGT, 5.8 million had IFG, 11.9 million had prediabetes, and 3.0 million had IGT and IFG [53].

### The genetics of type 2 diabetes and obesity

The search of human genetic factor(s) that predispose to type 2 diabetes and obesity has gone through two directions: by studying of rare mutations and by population-based gene-hunting for the primary causes [54-56]. It is generally accepted, after more than 20 years of intense effort, that environmental factors such as lifestyle and dietary composition play profound roles for the pathogenesis of type 2 diabetes and obesity. Furthermore, it is a consensus in the field that type 2 diabetes and obesity are polygenic diseases. As such, the population based gene-hunting efforts have not yielded conclusive "diabetogenes" and "obesitogenes". In contrast, through the candidate approach by studying the rare mutations, several genes have been identified as genes that are associated with type 2 diabetes and obesity. These genes and the studies are summarized in Table 2.

**Table 2:** Mendelian causes of type 2 diabetes and obesity

**Type 2 diabetes**		
Mutations	Gene Names	Reference
*ABCG8*	ATP-binding cassette Subunit C	[97, 98]
*CAPN10*	Calpain 10	[99, 100]
*GCGR*	Glucagon receptor	[101]
*GCK*	Glucokinase	[102]
*KCNJ11*	Potassium channel subunit J, member 11	[103]
*PPARG*	Peroxisome proliferator-activated receptor γ	[104]
*HNF4A*	Hepatocyte nuclear factor 4α	[105]
*HNF1A*	Hepatocyte nuclear factor 1α	[106]
*SLC2A1*	Glut 1	[107]
*INS*	Insulin	[108]
*INSR*	Insulin receptor	[109, 110]
*Mitochondrial genome*	Mitochondrial DNA	[111]

**Obesity**		
*Diabetes*	Leptin receptor	[112]
*Mc4r*	Melanocortin-4 receptor	[113]
*Obese*	Leptin	[114]
*PCSK1*	Prohormone convertase 1	[115]
*Pomc*	Pro-opiomelanocortin	[116]
*SLC6A14*	Solute carrier family 6 member 14	[117]

## Prevention of type 2 diabetes

Because of the devastating negative economic impact and human cost of type 2 diabetes, it is highly desirable that this disease can be prevented. Proof-of-concept studies for the prevention of type 2 diabetes have been conducted in several clinical trials in recent years. The outcome of these trials provide convincing evidence that either through lifestyle adjustment or through pharmacological treatment, type 2 diabetes can be prevented or, at least, be delayed. This conclusion is of profound implication in our way of thinking about the diabetes treatment and about the direction of finding new pharmacological entity as well as policy changes in the health care system.

There are four randomized controlled trials reported to-date for lifestyle modification. The conclusions from these studies are in general supportive of each other, and strongly argue for lifestyle modification as an effective way to prevent type 2 diabetes. The first study, Da Qing Study, dates back to 1986 [57]. Individuals diagnosed with IGT (577 patients) were randomized either to a control group or to one of three active treatment groups: diet only, exercise only, or diet plus exercise. Follow-up evaluation examinations were conducted at 2-year intervals over a 6-year period to identify subjects who developed type 2 diabetes. The cumulative incidence of diabetes at 6 years was 67.7%, 43.8%, 41.1%, and 46.0% in the control group, diet group, exercise group, and diet-plus-exercise group, respectively. After adjustment of BMI difference, the diet, exercise, and diet-plus-exercise interventions were associated with 31%, 46%, and 42% reductions in risk of developing diabetes, respectively.

The second study was conducted in Finland. In the Finnish trial [58], 522 middle-aged, overweight with impaired glucose tolerance subjects were randomly assigned to either the intervention group or the control group. The intervention group received individualized counseling aimed at reducing weight, total intake of fat, and intake of saturated fat and increased intake of fiber and physical activity. During the 3.2 years of follow-up, the cumulative incidence of diabetes was 11% which was reduced by 52% compared to the control group with an incidence of 23%. In this time period, the risk of diabetes was reduced by 58% in the intervention group.

In the third study, the Diabetes Prevention Program [59], a direct comparison was made between the lifestyle modification versus medication with metformin. Nondiabetic candidates (3234) with elevated fasting and post-load plasma glucose concentrations were randomized into placebo, metformin (850 mg twice daily), or a lifestyle-modification program (with the goals of at least a 7 percent weight loss and at least 150 minutes of physical activity per week). After a follow-up of 2.8 years, the incidence of diabetes was 11.0%, 7.8%, and 4.8% each year in the placebo, metformin, and lifestyle groups, respectively. The lifestyle intervention reduced the incidence by 58% and metformin by 31%, as compared with placebo; the lifestyle intervention was significantly more effective than metformin.

More recently, a fourth study comparing lifestyle modification and metformin medication was conducted in India [60]. A cohort of 531 subjects with IGT were randomized into four groups: control, lifestyle modification, metformin, lifestyle modification in addition to metformin. Diabetes developed in 49.6%, 35%, 39.8% and 34.7% respectively after 30 months of follow-up. This study adds another piece of evidence that lifestyle modification is effective in preventing diabetes. It also concluded that the lifestyle modification effect is not enhanced by metformin.

The success of these major diabetes prevention trials has impacted the way of approaching diabetes care. The ADA has recommended that all overweight people, aged ≥ 45 years with prediabetes be considered potential candidates for diabetes prevention. Overweight younger individuals with prediabetes and other risk factors should also be inducted into a diabetes prevention program [61].

## Lifestyle modification

The success of the DPP and other trials provide compelling evidence that modification of lifestyle, including increasing exercise along with decreased caloric intake, is an effective way of preventing or delaying the onset of type 2 diabetes in high risk populations. Interestingly, the pharmacological agent, metformin, only provided a modest effect in the DPP trial. Furthermore, there was no additional benefit of metformin over the lifestyle modification in its effect on diabetes prevention. Since the metformin mechanism of action might be through activation of AMPK [62], a kinase that is activated during exercise [63], it is possible that exercise may act through the same molecular cellular pathway and therefore there are no additive effects of these two treatment regimens.

One important aspect of combating the epidemics of obesity and type 2 diabetes has been through dietary strategy. Recently, very-low-carbohydrate diets have gained much popularity [64-67]. These diets have produced results at least comparable to and frequently better than traditional diets [68-70] although their acceptance among official agencies is limited at best. More recently the effects of low-carbohydrate/high-protein diets on the blood glucose levels, insulin resistance have been evaluated in obese/over-weight type 2 diabetes patients. One study comprised inpatient comparisons of a low-carbohydrate diet (20 g carbohydrate per day and unlimited protein and fat) among 10 obese patients with type 2 diabetes versus another 10 patients in a usual diet for 14 days. On the low-carbohydrate diet, mean energy intake decreased from 3111 kcal/d to 2164 kcal/d. This resulted in a mean weight loss of 1.65 kg, decrease in hemoglobin A_1c _from 7.3% to 6.8%, and insulin sensitivity improvement by about 75% [71]. The other study used a moderately low carbohydrate diet with a carbohydrate:protein:fat ratio of 20:30:50. Ingestion of this diet for 5 weeks in patients of untreated type 2 diabetes decreased hemoglobin A_1c _from ~9.8% to ~7.6% [72]. These study seem to indicate, by solely decrease the carbohydrate intake, it empowers patients with diabetes to ameliorate hyperglycemia without pharmaceutical intervention. Because glucose is the major insulin secretagogue carbohydrate reduction would be expected to be beneficial in type 2 diabetes and the use of such diets has been summarized by Arora & McFarlane [64] although, as noted above, official recommendations generally continue to recommend low fat and high carbohydrate intake.

## Medications in diabetes prevention

Although lifestyle modification is the most effective way of preventing diabetes in the clinical trials, the implementation demands a high level of discipline for the patients, which may preclude its effectiveness in the general population. In the long run, safe, effective medications, therefore, will likely be the best choice for intervention. One important lesson from the success of prevention trials is that lifestyle modification that results into the weight change has proven to disrupt the apparent pathological connection of overweight and the development of type 2 diabetes. Any drug that reduces weight increases the disposal of excess energy, or mimics exercise could be an effective treatment for diabetes prevention. The potential non-pharmaceutical approach and the pharmaceutical approach of diabetes prevention are summarized in table  3.

### Anti-obesity drugs

The concept of using an anti-obesity strategy to prevent diabetes was dramatically demonstrated in the follow-up studies for clinically severe obese (> 45 kg excess body weight) patients that received bariatric surgery [73]. During the ~5 year follow-up, among the experimental group of patients that included 109 patients with IGT who underwent bariatric surgery for weight loss, only 1 patient developed diabetes, resulting in a conversion rate of only 0.15 cases per 100 person-years. This rate is 30 fold lower than that found in the control group where the rate was found to be 4.72 cases per 100 person-years. In another prospective study conducted in Sweden, the Swedish Obese Subjects (SOS) study, compared the incidence of development of diabetes for 346 patients receiving gastric bypass surgery to the same number of obese control subjects receiving "conventional nonpharmacological obesity treatment" [74]. For these two sets of clinically obese patients, during the 8-year follow-up, the conventional nonpharmacological obesity treatment, in the hands of a primary health care system, had no effect on body weight, whereas gastric bypass surgery resulted in 18- to 30-kg maintained weight loss. This intentional weight loss in severely obese individuals reduces the 8-year incidence of diabetes by 5-fold. In addition to the benefit illustrated in the dramatic weight loss, modest weight control also added the benefit for diabetes prevention [75,76].

Orlistat (Xenical) is one of the few existing anti-obesity drugs in the market. In 2004, after the completion of a double-blind, placebo-controlled prospective study known as Xenical in the Prevention of Diabetes in Obesity Subjects (XENDOS), the European Commission approved labelling for the reduction of risks associated with the development of type 2 diabetes. In this study, 3,305 obese patients were followed over a 4-year period. Patients who received orlistat not only lost more weight, compared with those receiving placebo (5.7 kg vs. 3.0 kg), but also exhibited a 37% reduction in the incidence of type 2 diabetes during the treatment period [77]. Additional data indicated that orlistat reduced the doses of antidiabetic drugs by 23% during the treatment [78]. However, the profound gastrointestinal negative side effects of orlistat (such as oily spotting, flatus with discharge, fecal urgency) cause inconsistent compliance. This greatly reduces the use of this anti-obesity drug for the prevention of diabetes.

Rimonobant, the first selective cannabinoid type 1 (CB1) receptor antagonist, developed as an anti-obesity agent, is nearly completing phase III clinical trials. Originally, it was assumed that a CB1 antagonist would primarily reduce the food intake through a central nervous system-mediated effects [79,80]. Recent emerging evidence in experimental animals clearly indicate that the reduction of body weight and fat mass effected by CB1 antagonists is also due to peripheral mechanisms [81]. These may include a decrease in lipogenesis [82] and an increase in fatty acid oxidation [83]. The data from clinical trials of rimonobant further suggests that the weight loss, derived from the combined anorectic effect and the peripheral healthy effects in modulating fat metabolism, may have benefit for the treatment of Metabolic Syndrome. Rimonabant in Obesity Europe (RIO Europe) is the first of four Phase III studies whose partial data have been revealed [84]. Results of the RIO Europe program indicate that rimonabant at 20 mg/day, is associated with a significant decrease in body weight, as well as with a substantial mobilization of abdominal adipose tissue as indicated by a considerable reduction in waist circumference. Furthermore, the administration of rimonabant produced beneficial metabolic effects on plasma lipid parameters and on the glycemic profile, including the improvement in insulin sensitivity. Thus, these improvements decrease several parameters which comprise the metabolic syndrome in the patient population. As RIO Europe is continuing, it is much anticipated that rimonabant treatment will have significant effect on reducing the incidence of type 2 diabetes among the ~1000 patients with BMI ≥ 30 kg/m^2^.

### Other mechanisms for diabetes prevention

In the original design of the DPP trial, troglitazone, a synthetic agonist of peroxisome proliferator-activated receptor (PPAR)-γ, known for its insulin-sensitizing effect, was included as an independent therapeutic arm [85]. Due to the concern of liver toxicity of troglitazone, this arm was discontinued in 1998 [86]. Using the "short-term" data derived from the mean 0.9 year of troglitazone treatment, the diabetes incidence rate was estimated to be 3.0 cases/100 person-years, compared with 12.0, 6.7, and 5.1 cases/100 person-years in the placebo, metformin, and lifestyle modification participants [86]. The delay of diabetes by troglitazone was associated with preservation of β-cell compensation for insulin resistance [87]. The therapeutic benefit of troglitazone appeared to be reversible. During the 3 years after troglitazone withdrawal, the diabetes incidence rate bounced back to a level similar to the placebo group [86]. According to these data, insulin sensitization through a PPAR-γ activation mechanism is effective in diabetes prevention. However the availability of an agent with an appropriate safety property remains to be a challenge for long term and wide uses.

Acarbose, an α-glucosidase inhibitor, that inhibits the hydrolysis of non-absorbable oligosaccharides and polysaccharides into absorbable monosaccharides which takes place in the brush border of enterocytes, is another agent that is efficacious in reducing blood glucose and insulin level, especially in the postprandial conditions. In the STOP-NIDDM trial, the effect of Acarbose in preventing or delaying the development of type 2 diabetes was assessed [88]. In this multicenter, placebo-controlled randomised trial, patients with IGT were randomly allocated to 100 mg acarbose or placebo three times daily. After a mean follow-up of 3.3 years, 32% patients randomised to acarbose and 42% randomised to placebo developed diabetes. Furthermore, acarbose significantly increased reversion of impaired glucose tolerance to normal glucose tolerance. It was concluded that Acarbose could be used, either as an alternative or in addition to changes in lifestyle, to delay development of type 2 diabetes in patients with IGT.

If preservation of β-cell compensation is an effective way to prevent the progression of type 2 diabetes, strategies based on glucagons-like peptide (GLP) -1 should be successful as well. GLP-1 is an incretin hormone stimulating the glucose-induced insulin secretion in pancreatic β-cells [89]. It not only stimulates the biosynthesis and exocytosis of insulin, but it also has effects on β-cell growth and survival that lead to increased β-cell mass. Moreover, GLP-1 also inhibits the motility in the gastrointestinal tract which slows down the passage of nutrients to the small intestine. When administered in peripheral, GLP-1 also increases the satiety level [90]. In the clinic, a synthetic GLP-1 receptor agonist with a pro-longed half-life compared to the endogenous GLP-1 significantly improved HbA_1C _[91] along with obvious weight reduction in the patients [92]. Inhibitors of dipeptidyl peptidase (DPP) IV, a protease responsible for the degradation of short-lived endogenous GLP-1, not only extended the half-life of this incretin hormone, but also reduced the excursion of glucose in diabetic patients [93]. These data demonstrate that GLP-1 based therapies, either through administration of exogenous synthetic GLP-1 receptor agonists or through inhibiting DPP IV, are effective in diabetes treatment. Whether these strategies will be successful in pre-diabetes patients to prevent or to delay the onset of type 2 diabetes, awaits more clinical studies designed to address the issue.

## Concluding thoughts

Both the diabetes and the obesity epidemics have posed serious assaults on health care. It is anticipated that it will be an uphill battle to fight these ever increasing epidemics, especially since, individually, these conditions are difficult to treat. However, considering the history in the advances of modern medicine, the future looks brighter. The pharmaceutical breakthrough of the statin-class of safe and effective drugs for plasma cholesterol control has caused the mortality rate for cardiovascular disease to decrease [94,95]. The economic benefits have also been demonstrated [96]. Today, there is wide public awareness that obesity and a sedentary lifestyle are culprits leading to the development of diabetes. It is generally known that modifying the sedentary lifestyle with exercise can have a positive impact on diabetes. There are also active efforts in modulating the dietary composition, particularly through cutting down carbohydrates, in the public. What is lacking presently is a true breakthrough for a safe and effective way to treat weight problems. The good news is that there are many appetite suppressant drugs and other anti-obesity drugs in discovery pipelines of the pharmaceutical industry. As more of these future drugs move forward in the pipeline and eventually to the market, it is possible to be optimistic that an era of type 2 diabetes prevention may be about to begin.

**Table 3:** Potential therapies for the prevention of type 2 diabetes.

**Non-pharmaceutical Approach**			
Method			Clinically Proven

Lifestyle modification			yes
Bariatric surgery			yes

**Pharmaceutical Approach**			

Drugs	Molecular Target	Site(s) of Action	Clinically Proven

Metformin	Unknown	Liver (muscle)	Yes
Acarbose	α-glucosidase	Intestine	Yes
Olistat	lipase	Intestine	Yes
Thiazolidinediones	PPARγ	Liver, fat, muscle	Yes
Rimonobant	CB-1	Brain (fat, liver)	No
DPP4 inhibitors	DPP4	pancreatic islet beta-cells	No
GLP1 analogs	GLP1-receptor	pancreatic islet beta-cells	No
